# Testing the relationships of hope and negotiable fate with sleep quality among university students in Hong Kong

**DOI:** 10.3389/fpsyg.2024.1503036

**Published:** 2025-01-08

**Authors:** Hok I. Chan, David B. Feldman, Tse-Mei Shu

**Affiliations:** ^1^Department of Counseling Psychology, Santa Clara University, Santa Clara, CA, United States; ^2^Division of Social Science, Hong Kong University of Science and Technology, Hong Kong, Hong Kong SAR, China

**Keywords:** hope, negotiable fate, sleep, stress, university

## Abstract

For university students, who are often under significant stress to perform well, sleep is particularly important, playing roles in learning and academic performance. Research demonstrates a relationship between hope and sleep quality. However, most work concerns Western samples, and the hope construct has been criticized for a Western bias. No research investigates sleep quality’s relationship to “negotiable fate,” a construct more relevant to Asian cultures. Whereas hope involves the individualistic expectancy that goals are achievable through personal action, negotiable fate involves the more collectivist-informed belief that control can be exercised over goals within limits of fate (family, community, society, other external factors). We investigated the relationship of hope and negotiable fate with sleep quality in 160 university students in Hong Kong, administering measures of hope, negotiable fate, sleep quality, depression, anxiety, stress, and life satisfaction. Lower hope was associated with poorer self-reported sleep quality. In addition, consistent with past research, mental health variables (particularly stress) accounted for significant variance in this relationship. Negotiable fate, however, did not relate to sleep quality.

## 1 Introduction

Sleep quality is an important indicator of mental and physical health (Medic et al., 2017). Wong and colleagues, for instance, performed a longitudinal study of university students in Hong Kong and Macau, finding that sleep difficulties predicted poorer academic performance, physical health, and psychological health (Wong et al., 2013). A recent meta-analyses also indicates relationships between sleep disturbances and suicidal behavior (Baldini et al., 2024). For university students, sleep is particularly significant, as it plays a crucial role in various facets of learning, including memory, information processing, and cognitive functioning (Gobin et al., 2015; Killgore, 2010; Krause et al., 2017). Therefore, it is important to identify factors associated with sleep quality in students. We examine two such factors: hope and negotiable fate.

Existing research demonstrates a relationship between hope and sleep quality (Lau et al., 2017; Mystakidou et al., 2009; Mansano-Schlosser et al., 2017; Winsler et al., 2015; Rubens et al., 2020). Additionally, hope appears to be associated with negotiable fate (Au and Savani, 2019; Yau and Shu, 2024). Nevertheless, to our knowledge, no study has yet investigated the relationship between negotiable fate and sleep quality. Moreover, the studies just cited have almost exclusively involved samples from the United States or Europe, with only one including an Asian sample (i.e., Lau et al., 2017), limiting the generalizability of findings across cultures. Given that sleep quality as well as beliefs and habits regarding sleep vary across cultures (Arslan et al., 2015), studying these relationships in an Asian sample may help clarify whether hope functions similarly in this context as a predictor of sleep quality.

In this article, we report a study exploring the relationships of hope and negotiable fate with sleep quality in a sample of Hong Kong university students. We also investigate the possible roles of depression, anxiety, stress, and life satisfaction in accounting for variance in these relationships. Next, we define hope and negotiable fate and review the literature connected with both constructs.

## 2 Hope

According to Snyder’s Hope Theory (Snyder et al., 1991; Snyder, 2002), the most researched model of hope during the past three decades, hope is conceptualized as a trait-like goal-directed cognitive construct. Hope integrates two components: agency thinking and pathways thinking. Agency thinking refers to individuals’ beliefs regarding their capacity to pursue goals and persist in the face of obstacles. Agency thoughts such as “I can do this,” or “I can achieve my goals, even when things seem hard,” provide motivation. Pathways are plans, strategies, or routes to achieving desired goals. Thus, people engage in pathways thinking whenever they envision ways to reach their desires.

Research demonstrates that higher hope is a robust predictor of goal accomplishment among college students (Feldman et al., 2009). Higher hope also is consistently linked with better school outcomes (Feldman and Kubota, 2015; Ge et al., 2023; Rand et al., 2011; Rubens et al., 2020). The cognitive nature of Hope Theory involves individuals’ beliefs in their capacity to generate routes to goals (i.e., pathways) and motivate themselves to pursue those goals (i.e., agency). Thus, when students are hopeful, they are more likely to engage in setting and pursuing academic goals.

Most relevant to the present study, research links hope and sleep quality (Lemola et al., 2011; Rubens et al., 2020). One study found that greater hope was related to lower depression and better sleep quality in women with breast cancer (Mansano-Schlosser et al., 2017). Likewise, in Latinx middle schoolers in the United States, hope was negatively associated with daytime sleepiness (Rubens et al., 2020). Additionally, in a sample of middle-school and high-school students in Fairfax County, Virginia, greater hopelessness was associated with lower sleep duration (Winsler et al., 2015). It should be noted, however, that hopelessness is a somewhat different construct than hope (see Snyder et al., 2001) and, in the latter study, hopelessness was measured with a single face-valid item.

Of note, mental health variables may account for variance in the relationship between hope and sleep quality, and thus statistically controlling for such variables appears to attenuate the hope-sleep relationship. Uchino et al. (2017) found that depressive symptoms and life satisfaction statistically accounted for this relationship among healthy adult participants in the United States. Similarly, Mystakidou et al. (2009) found that depression accounted for variance in the relationship between poorer sleep quality and hopelessness among Greek patients with terminal cancers. Stress may also play a role. Researchers have observed that Hong Kong, in particular, has a highly competitive educational environment, which may result in stress and sleep difficulties (Ng et al., 2016; Tsang and Lian, 2020; Tsui and Wing, 2009). Examining hope as a potentially protective factor against sleep issues in this context may have practical implications for improving student well-being and resilience. Also in this regard, despite the evidence linking hope and sleep, research is needed in individuals outside the Western context.

## 3 Negotiable fate

Critics have raised concerns about Hope Theory’s conceptualization as an individualistic construct focusing on personal motivation and planning (Bernardo, 2010; Du and King, 2013). This perspective may not fully resonate with collectivist cultures such as those in East Asia. Instead, the construct of negotiable fate may hold greater significance (Au et al., 2012). According to negotiable fate, individuals can pursue goals and influence outcomes within the limits of fate (Au and Savani, 2019). External factors such as socio-cultural environments (e.g., family, community, society), socio-economic status, and uncontrollable life events create constraints. However, how people act within these constraints shapes their lives. Unlike hope, negotiable fate acknowledges the interconnectedness of individuals within social and cultural contexts.

Like hope, negotiable fate has been linked to both academic performance and goal achievement in university students and other populations. Yau and Shu (2023) found that it was associated with school engagement in undergraduates in Hong Kong. In another university sample in Romania, researchers found that negotiable fate beliefs were associated with greater career-goal-setting self-efficacy (Kotta et al., 2021). Moreover, in a study of executives at numerous firms in China, negotiable fate was related to individuals’ levels of entrepreneurial orientation as well as their firms’ financial and innovative performance (Au et al., 2017).

To our understanding, no published work yet investigates negotiable fate’s relationship to sleep quality. Given that negotiable fate appears to align more closely with collectivist cultural concepts and is perhaps more applicable to Asian culture, it seems important to examine its relationship with sleep quality among students with these cultural backgrounds.

## 4 The present study

In the current research, we investigated the relationships of hope and negotiable fate with sleep quality among university students in Hong Kong. We also investigated the degree to which depression, anxiety, stress, and life satisfaction may account for variance in this relationship. Unlike previous research conducted in U.S. or European samples, this study fills a gap in the literature by investigating these variables in an Asian cultural context. This is also the first investigation of the relationship between negotiable fate and sleep quality.

Specifically, we conducted a cross-sectional survey of sleep quality, hope, negotiable fate, mental health symptoms (depression, anxiety, and stress), and life satisfaction. We propose three hypotheses based on previous research: First, we expect that hope and negotiable fate will be positively correlated with one another, given their mutual focus on goal pursuit. Second, we hypothesize positive relationships between hope and sleep quality as well as between negotiable fate and sleep quality. Third, we hypothesize that depression, anxiety, stress, and life satisfaction will individually and collectively account for variance in these relationships.

## 5 Method

### 5.1 Participants

Participants were 160 students from a large, public university in Hong Kong who took part as a means of fulfilling the research participation requirements of their psychology courses. Participants indicated age categorically (by range), with the mode being 18–24, reported by 99.4% (*n* = 159) of students. In terms of gender, 61.9% (*n* = 99) identified as women, 36.9% (*n* = 59) as men, and 1.3% (*n* = 2) as gender non-binary. The vast majority of participants identified as Asian (93.1%, *n* = 149), with 4.4% (*n* = 7) identifying as White, and 2.5% (*n* = 4) preferring not to say.^1^ Regarding year in school, 2.5% (*n* = 4) were first year students, 8.8% (*n* = 14) second year, 26.3% (*n* = 42) third year, 41.9% (*n* = 67) fourth year, and 20% (*n* = 32) fifth year and beyond, with 1 participant declining to state.

### 5.2 Procedure

Data were collected as part of a larger study examining the relationship between cultural beliefs and sleep quality in Hong Kong and the United States. Participants signed up for the study via an online platform (i.e., Sona Systems, 2024) and were provided a link to our survey, which was administered via the Qualtrics (2024) online survey software. Once they agreed to the informed consent, they completed measures in a random order. The study was approved by the IRBs of the authors’ institutions.

### 5.3 Measures

#### 5.3.1 Adult hope scale (AHS)

The AHS (Snyder et al., 1991) is a self-report measure of dispositional hope. It contains 12 items with 4 tapping pathways thinking, 4 tapping agency thinking, and 4 serving as distracters. Example items include “I can think of many ways to get the things in life that are most important to me” (pathways), and “I energetically pursue my goals” (agency). Participants rate each item on a scale ranging from 1 (*definitely false*) to 8 (*definitely true*). Higher scores indicate greater levels of hope. Researchers have provided evidence to support the reliability and validity of the Hope Scale (Snyder et al., 1991). In the present study, the Cronbach’s alpha of the AHS was 0.90.

#### 5.3.2 Negotiable with fate measure (NFM)

The NFM (Au and Savani, 2019) is a self-report measure of the degree to which people believe they can exercise agency to navigate boundaries imposed by fate. It contains 10 items. Example items include “There’s no situation bestowed by fate that I cannot improve upon,” and “My fate can be changed for the better if I work hard at everything I do.” Participants rate each item on a scale ranging from 1 (*strongly disagree*) to 6 (*strongly agree*). Higher scores indicate greater perceptions of negotiable fate. Researchers have provided evidence to support the reliability and validity of the NFM (Au and Savani, 2019). In the present study, the Cronbach’s alpha of the NFM was 0.90.

#### 5.3.3 Pittsburgh sleep quality index (PSQI)

The PSQI (Buysse et al., 1989) is a self-report measure of sleep quality over the past month. It consists of 19 items, which combine to form seven components: subjective sleep quality, sleep latency, sleep duration, habitual sleep efficiency, sleep disturbances, use of sleep medication, and daytime dysfunction. Each component is scored from 0 (no difficulty) to 3 (severe difficulty), with the sum of these scores yielding one global index. In the present study, we utilized this global index. Example items include “During the past month, how would you rate your sleep quality overall?” and “During the past month, how often have you had trouble staying awake while driving, eating meals, or engaging in social activity?” Responses are provided on Likert-type scales, with anchors differing among items. Higher scores indicate poorer sleep quality. The validity and reliability of the PSQI have been well-documented (Buysse et al., 1989; Carpenter and Andrykowski, 1998). In the present study, the Cronbach’s alpha of the PSQI was 0.59.

#### 5.3.4 Depression, anxiety, stress scale (DASS-21)

The DASS-21 (Lovibond and Lovibond, 1995) is a self-report measure of mental health symptoms. It contains 21 items divided into subscales for depression, anxiety, and stress. Example items include “I could not seem to experience any positive feeling at all” (depression), “I was worried about situations in which I might panic and make a fool of myself” (anxiety), and “I found it difficult to relax” (stress). Participants rate each item regarding their experience over the past week on a 0 (*did not apply to me at all*) to 3 (*applied to me very much, or most of the time*) scale. Higher scores indicate greater symptoms. Research supports the reliability and validity of the DASS-21 (Antony et al., 1998). In the present study, Cronbach’s alphas for the DASS-21 were 0.90 for depression, 0.80 for anxiety, and 0.84 for stress.

#### 5.3.5 The satisfaction with life scale (SWLS)

The SWLS, developed by Diener et al. (1985), is a measure of overall life satisfaction. It contains 5 items, each presenting a statement about life. Sample items include “In most ways my life is close to my ideal,” and “I am satisfied with my life,” which participants rate on a scale from 1 (*strongly disagree*) to 7 (*strongly agree*). Higher scores indicate greater life satisfaction. Researchers have provided evidence for the SWLS’s reliability and validity (Diener et al., 1985). In the present study, Cronbach’s alpha for the SWLS was 0.89.

### 5.4 Statistical analyses

Demographic data were summarized using descriptive statistics (e.g., counts, percentages), and t-tests and ANOVAs were used to test for demographic differences on all study variables. All variables were also initially examined for skewness and kurtosis. We then used Pearson correlations to test for bivariate relationships. Finally, to test for the degree to which depression, anxiety, stress, and/or SWLS scores would account for variance in the relationship between AHS and PSQI scores, we performed a series of analyses using the PROCESS 4.2 macro (Hayes, 2018) within the SPSS 29.0 software package.

## 6 Results

Variables were first examined for assumptions of normality (i.e., kurtosis <4 and skewness <2; Boos and Hughes-Oliver, 2000). All variables met these assumptions, with skewness ranging from 0.028 to 0.783, and kurtosis ranging from −0.621 to 0.547. We also examined variables for demographic differences. For gender, we ran a series of t-tests comparing individuals who identified as women and those who identified as men (two participants who identified as gender non-binary were not included in this comparison to avoid violating statistical assumptions). No gender differences were found for AHS (*t* (156) = 1.45, *p* = 0.15), NFM (*t* (156) = 0.30, *p* = 0.76), PSQI (*t* (156) = 1.04, *p* = 0.30), SWLS (*t* (156) = −0.13, *p* = 0.89), DASS-21 depression (*t* (156) = 0.292, *p* = 0.77), DASS-21 anxiety (*t* (156) = 0.92, *p* = 0.36), or DASS-21 stress (*t* (156) = 0.474, *p* = 0.64). For year in school, we ran a series of between-subjects ANOVAs, also finding no mean differences on AHS (*F* (4, 154) = 0.87, *p* = 0.49), NFM (*F* (4, 154) = 0.1.08, *p* = 0.37), PSQI (*F* (4, 154) = 1.69, *p* = 0.16), SWLS (*F* (4, 154) = 0.73, *p* = 0.57), DASS-21 depression (*F* (4, 154) = 1.026, *p* = 0.40), DASS-21 anxiety (*F* (4, 154) = 0.60, *p* = 0.66) or DASS-21 stress (*F* (4, 154) = 1.49, *p* = 0.21). Given the lack of variability in race and age in our sample, no comparisons were run for these two variables.

Intercorrelations are presented in Table 1. As can be seen, the AHS and NFM were relatively strongly correlated (*r* = 0.63, *p* < 0.001). We furthermore found a negative correlation between AHS and PSQI scores (*r* = −0.18, *p* = 0.02), indicating that greater hope was associated with better sleep quality. No significant relationship was found between NFM and PSQI scores, *r* = −0.11, *p* = 0.18. As such, we used only the AHS in subsequent analyses.

**Table 1:** Intercorrelations.

Variable	1	2	3	4	5	6
1. Hope	–	0.63**	0.18*	0.54**	0.24**	0.36**
2. Negotiable fate		–	−0.11	0.42**	−0.14	−0.15
3. Sleep quality			–	0.35**	0.39**	0.43**
4. Depression				–	0.68**	71**
5. Anxiety					–	77**
6. Stress						–

*p < 0.05, **p < 0.01.

**Figure 1:**
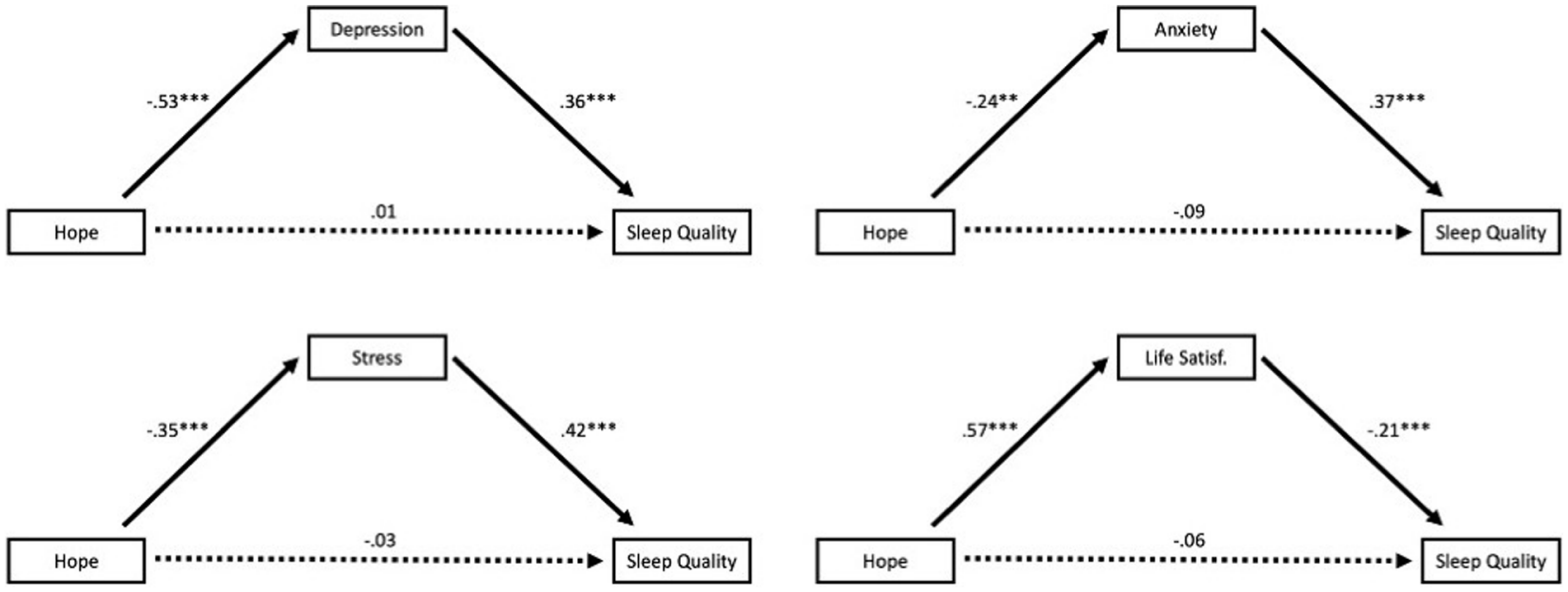
Models with standardized coefficients. **p < 0.01, ***p < 0.001.

**Figure 2:**
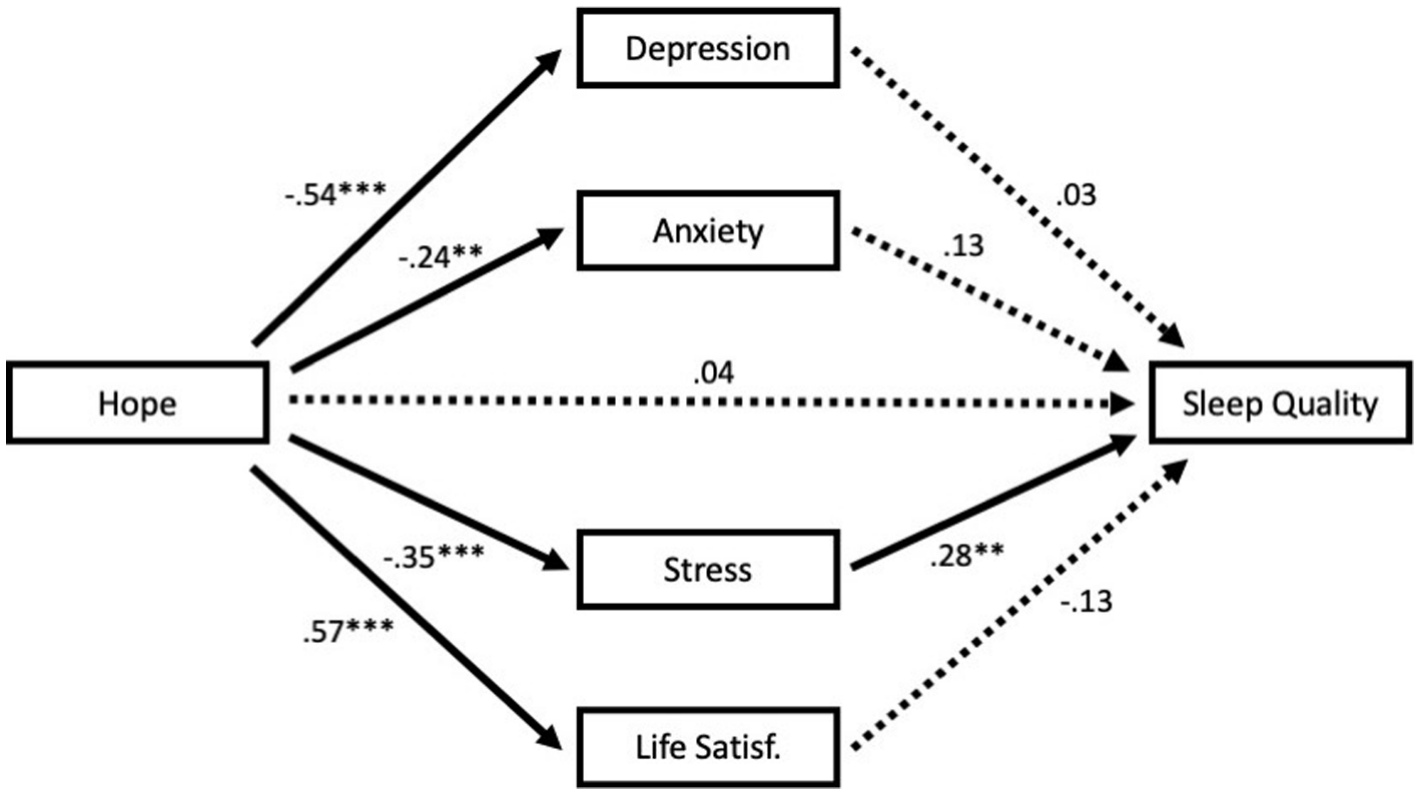
Model including depression, anxiety, stress and life satisfaction entered in parallel. Coefficients are standardized. **p < 0.01, ***p < 0.001.

As previously stated, we hypothesized that depression, anxiety, stress, and/or SWLS scores would account for variance in the relationship between AHS and PSQI scores. That is, including such variables in our analysis should statistically attenuate the association between hope and sleep quality. Thus, using the PROCESS macro (Model 4; Hayes, 2018) in SPSS, we ran four separate analyses. In each, AHS served as X variable, PSQI as Y variable, and one of the following as M variable: DASS-21 depression, DASS-21 anxiety, DASS-21 stress, or SWLS. Each of these four variables fully accounted for the sleep-hope relationship, effect = −0.19, LLCI = −0.32, ULCI = −0.09 (for depression); effect = −0.09, LLCI = −0.18, ULCI = −0.02 (for anxiety); effect = −0.15, LLCI = −0.23, ULCI = −0.07 (for stress), effect = −0.12, LLCI = −0.23, ULCI = −0.02 (for life satisfaction). Figure 1 contains these models with standardized beta weights. Lastly, to determine which of these variables were “essential” in accounting for variance in the AHS-PSQI relationship, we ran a final analysis with depression, anxiety, stress, and life satisfaction entered simultaneously (i.e., in parallel; see Figure 2). Only stress emerged as accounting for variance in this relationship, effect = −0.10, LLCI = −0.19, ULCI = −0.01.

## 7 Discussion

In the present study, we examined the relationships of hope and negotiable fate to sleep quality among university students in Hong Kong. As hypothesized, we found that hope and negotiable fate were positively and relatively strongly correlated with one another. This relationship is consistent with previous research (Yau and Shu, 2024) and is not surprising given these two constructs’ mutual focus on goal pursuit. Also as hypothesized, we found a positive relationship between hope and better sleep quality. Finally, we found that depression, anxiety, stress, and life satisfaction each accounted for variance in this relationship. When examining all four of these variables concurrently, however, stress stood out as the only one uniquely accounting for variance in the hope-sleep relationship.

Despite past theorizing that negotiable fate is more consistent with collectivist cultures, sleep quality was unrelated to negotiable fate in the present sample. This outcome is surprising given the cultural context of Hong Kong, which is traditionally understood to be relatively collectivistic (Hofstede, 1980). However, some research shows that people in China, more broadly, appear to be increasingly prioritizing individualist factors in evaluating and understanding their lives (Steele and Lynch, 2013; Yan, 2010). It may also be that, regardless of the broader cultural context, sleep is perceived to be an activity more under individual control, thus rendering hope the more relevant construct. Future research in other Asian nations with diverse cultural values may help shed additional light.

### 7.1 Limitations

As with any study, the current research has limitations. First, our study was cross-sectional, limiting causal conclusions. One possible explanation for our findings is that hope reduces stress, thereby benefitting sleep quality. Alternatively, sleep may also reduce stress, thus increasing hope. Future studies with experimental or longitudinal designs may be important in clarifying the directionality of these relationships. Second, our sample consisted of college students in Hong Kong. Appropriate caution should thus be taken in generalizing the results to other age groups and other populations across regions, nations, or cultures, including within Asia. Beliefs and norms regarding sleep habits and hope vary across cultures, and beliefs or attitudes toward sleep may be associated with sleep duration (Arslan et al., 2015). Thus, researchers should consider replicating our results in diverse samples. Finally, this study utilized a self-report measure of sleep quality (Pittsburgh Sleep Quality Index). Although widely used, it is potentially subject to social desirability and memory recall biases, similar to all such instruments. Moreover, in the present sample, this measure had somewhat lower-than-optimal internal consistency (*alpha* = 0.59), though it should be noted that the distribution of scores appeared normal and its relationships with other variables were consistent with past research (e.g., Lau et al., 2017; Mansano-Schlosser et al., 2017; Uchino et al., 2017). Nonetheless, future researchers should consider replicating the present study using more objective sleep measures (e.g., actigraphy).

### 7.2 Implications and conclusion

Sleep is an important factor for academic performance and cognitive development (Gobin et al., 2015; Krause et al., 2017), and hope is consistently related to sleep quality (Lemola et al., 2011; Rubens et al., 2020). As mentioned, we found that higher hope was associated with better sleep quality among university students in Hong Kong, and that mental health variables (especially stress) accounted for variance in this relationship. Recognizing hope, stress, and sleep as related and potentially modifiable factors, researchers should consider testing intervention programs emphasizing these factors using experimental designs. For instance, given that stress was found to uniquely account for variance in the relationship between hope and sleep quality, educational institutions and mental health organizations could prioritize stress management interventions. Implementing stress reduction programs, like mindfulness training or cognitive-behavioral strategies tailored to students, could potentially play a role in improving sleep quality and overall well-being.

Furthermore, given that higher levels of hope are associated with better sleep quality, educational policies could aim to foster a hopeful mindset. For example, incorporating goal-setting workshops or hope-centered counseling into student services could potentially be beneficial. Several such interventions already exist (see Cheavens and Whitted, 2023 for a review). For instance, Cheavens et al. (2006) developed an 8-session group hope intervention and tested it in a sample of adults in the United States with a variety of mental health diagnoses. Feldman and Dreher (2012) shortened and adapted this intervention for delivery in a single 90-min session with college students. Such interventions could be adapted and augmented with components explicitly addressing both stress and sleep.

In conclusion, we believe our study contributes to the evolving body of evidence on the relationship between hope and sleep. Specifically, we extended this exploration to college students in Hong Kong, augmenting our knowledge across cultures. We encourage researchers to continue expanding beyond Western cultures, diversify samples, and incorporate cultural contexts to better understand this relationship and assess the broader applicability of findings across global populations.

## Data Availability

The raw data supporting the conclusions of this article will be made available by the authors, without undue reservation.

## Footnotes

^1^We ran all subsequent analyses both including and excluding the 11 participants who did not indicate that their race/ethnicity was Asian. Because the results of these analyses were similar, we have included these 11 participants in the analyses reported here.
